# Inequity in the healthcare utilization among latent classes of elderly people with chronic diseases and decomposition analysis in China

**DOI:** 10.1186/s12877-022-03538-x

**Published:** 2022-11-11

**Authors:** Jie Zhao, Chaoyang Yan, Dan Han, Yunyi Wu, Hui Liao, Ying Ma, Mei Zhang, Sangsang Li, Jing Wang

**Affiliations:** 1 Department of Health Management, School of Medicine and Health Management, Tongji Medical College, Huazhong University of Science and Technology, Wuhan 430030, Hubei, China; 2 The Key Research Institute of Humanities and Social Science of Hubei Province, Huazhong University of Science and Technology, Wuhan 430030, Hubei, China; 3 Institute for Poverty Reduction and Development, Huazhong University of Science and Technology, Wuhan 430030, Hubei, China

**Keywords:** Healthcare utilization, Inequity, Concentration index, Latent class analysis

## Abstract

**Background:**

Studies have shown chronic disease-based healthcare utilization inequity is common. Hence, exploring this issue can help in establishing targeted measures and protecting the rights and interests of vulnerable groups. Against this background, the purpose of this study is to explore the latent classification of elderly patients with chronic disease and compare healthcare utilization inequity among latent classes.

**Methods:**

This study used the data of 7243 elderly patient with chronic diseases collected from the China Health and Retirement Longitudinal Study in 2018. Latent class analysis was used to classify the patients with chronic diseases, and analysis of variance and ${x}^{2}$ tests were utilized to test the differences in characteristics among latent classes. Healthcare utilization inequity was measured based on the concentration index (CI), and the CI was decomposed to compare the horizontal index of healthcare utilization among the latent classes.

**Results:**

The patients with chronic diseases were divided into five latent classes, namely, the musculoskeletal system, hypertension, respiratory system, digestive system and cardiovascular system groups. Statistically significant differences in social demographic characteristics were observed among the five latent classes (*P* < 0.05). A pro-rich healthcare utilization inequity for all respondents was observed (outpatient CI = 0.080, inpatient CI = 0.135), and a similar phenomenon in latent classes was found except for the musculoskeletal system group in outpatient visits (CI = -0.037). The digestive system group had the worst equity (outpatient CI = 0.197, inpatient CI = 0.157) and the respiratory system group had the best (outpatient CI = 0.001, inpatient CI = 0.086). After balancing the influence of health need factors, healthcare utilization inequity was almost alleviated. Furthermore, for all respondents, the contribution of health need factors (65.227% for outpatient and 81.593% for inpatient) was larger than that of socioeconomic factors (-21.774% for outpatient and 23.707 for inpatient), and self-rated health status was the greatest contributor (57.167% for outpatient and 79.399% for inpatient). The characteristics were shown in latent classes.

**Conclusions:**

Healthcare utilization inequity still exists in elderly patients with chronic diseases, and the specific performances of inequity vary among latent classes. Moreover, self-rated health status plays an important role in healthcare utilization inequity. Providing financial support to low-income patients with certain chronic diseases, focusing on their physical and mental feelings and guiding them to evaluate their health status correctly could be essential for alleviating healthcare utilization inequity among elderly patients with chronic diseases.

## Introduction

Healthcare utilization equity is a core objective for health care systems because it is an important approach for safeguarding health equity [1]. Equity in access to healthcare utilization is one social determinant of health and health equity, which are fundamental results of all social and economic policies [2, 3]. Basing health service utilization on needs and preferences rather than on an economic level is the key to addressing healthcare utilization inequity [4].

However, with economic growth and medical technology improvements, medical expenses have increased annually, and healthcare utilization inequity has also expanded [5]. A review of the literature revealed that although the poor have a higher demand for healthcare utilization than the rich, the rich have greater access to healthcare utilization than the poor [3, 5]. Many factors account for this large gap. Our present research focuses on determining the influencing factors of healthcare utilization equity and identifying vulnerable populations.

Healthcare utilization inequity has a tight relationship with sample characteristics. For example, the elderly have higher rates of healthcare utilization than other age groups [6–8]. Furthermore, not only are females less likely to take proven effective treatment than males, but they are also more likely to give up treatment [9, 10]. Economic income, education level, and other socio-economic factors are related to opportunities for accessing healthcare. In general, a higher socio-economic level is associated with increased opportunities to use healthcare [11–14]. High-income earners often use privately owned care resources in developed and developing countries [13, 14]. Healthcare utilization differences also exist among ethnicities in the same place: people of colour and ethnic minorities are usually disadvantaged [15, 16].

Healthcare utilization also exhibits regional differences. It usually tends to concentrate in cities and affluent areas [11, 17]. In Colombia, older adults residing in urban areas nearly have two times the odds of healthcare usage than rural residents [18]. However, the economic level is not the only reason for regional differences given that disparities in healthcare utilization can also be found in developed countries [19, 20].

Medical insurance is also important to healthcare utilization inequity. The uninsured are far more likely to forgo needed medical services than the insured [11, 21]. The National Health Insurance of Taiwan has helped increase healthcare utilization by the elderly [22]. However, disparities exist between people with different types of medical insurance, and are usually related to service coverage and financial support [5, 21, 23, 24].

Disease-based healthcare utilization discrepancy is considerably more common than other discrepancies, especially in chronic diseases. Numerous studies have found that healthcare utilization is usually concentrated in rich populations with chronic diseases [12, 25]. In Serbia, residents with non-communicable chronic diseases are three times more likely to make a general practitioner visit and approximately two times more likely to be hospitalized than others [26]. Similar findings can be obtained in many countries and places [27, 28].

Furthermore, current studies have revealed discrepancies in healthcare utilization among patients with different chronic diseases. A follow-up study compared healthcare utilization in patients with cardiovascular disease (CVD), chronic obstructive pulmonary disease (COPD), diabetes and mental disorders, and the results show that patients with diabetes had slightly more visits than others and that patients with COPD had longer hospital admission days [29]. Another study highlighted the differences in medication non-adherence with different chronic diseases, with the result that patients with osteoporosis and hyperlipidaemia had the worst medication adherence and patients with diabetes had the best [30]. In addition, patients with multiple chronic diseases used more healthcare services than patients with one chronic disease, and this feature is particularly evident in certain chronic disease combinations [31, 32]. Although studies have classified chronic disease combinations in different ways, combinations that include chronic kidney disease and mood disorders are often associated with more healthcare utilization [32–34].

Currently, scholars have conducted considerable research on the manifestations and causes of inequity in healthcare utilization, and have also found that the utilization of healthcare varies according to which chronic disease patients suffer, including the types and numbers of diseases. However, few studies have measured and compared healthcare utilization inequity among people with different types of chronic diseases, which is worth exploring. Determining which groups of patients with chronic diseases are more likely to experience healthcare utilization inequity will help in identifying targeted and comprehensive measures for each group. Moreover, according to the data of the World Health Organization (WHO) Study on global AGEing and adult health (SAGE), 74.5% of adults over 50 years old were identified as having a chronic disease, among which 39.9% had multiple chronic diseases [35]. Comparing the healthcare utilization inequities of chronic diseases is difficult because people with multimorbidities usually have complex needs for health care. In identifying differences in healthcare utilization inequity in chronic patients with chronic diseases and comorbidity or complications, latent class analysis (LCA) is suitable for segmenting the patients into latent classes with similar disease profiles, with each class containing individuals who are similar to each other and are different from individuals in other categories [36, 37]. Therefore, in this study, we aim to (1) explore the latent classification of elderly patients with chronic diseases, (2) calculate the concentration index (CI) and horizontal index (HI) to compare healthcare utilization inequities among latent classes and (3) decompose the CI to learn the contribution of each independent variables to the total inequity.

## Methods

### Data source

The data used for analysis in this study were collected by the China Health and Retirement Longitudinal Study (CHARLS) in 2018. CHARLS aims to set up a high-quality public micro-database that can provide a wide range of information ranging from socioeconomic status to the health status of residents aged 45 years and over. The CHARLS questionnaire contains basic information and behavioral data on the interviewees and their families, as well as personal health status and healthcare utilization. Using a stratified multi-stage PPS random sampling strategy, a total of 19,816 individual were sampled from 450 communities or villages in 150 counties or districts [38]. Thus, the data are highly representative.

The target population of this study is elderly patients with chronic diseases. Following the WHO’s definition of the elderly, we included 11,051 adults aged 60 years and above [39]. There were 8640 elderly patients with at least one chronic disease. The range of chronic diseases was determined by the CHARLS, and include hypertension, dyslipidemia, diabetes or high blood sugar, cancer or malignant tumor, chronic lung disease, liver disease, heart attack, stroke, asthma, kidney disease, digestive disease, mood disorders, memory-related disease, arthritis or rheumatism and asthma. The diseases were confirmed by the patient’s self-report of a physician’s diagnosis. After excluding the samples with missing relevant variables, such as healthcare utilization and demographic characteristics, the final sample size was 7,243.

## Variables and definitions

### Dependent variables

Healthcare utilization includes outpatient visits and inpatient visits. Therefore, our survey selected two indicators to measure healthcare utilization: frequency of one-month outpatient visits and frequency of one-year inpatient visits. Participants were asked the following: (1) How many times did you visit a doctor for outpatient care during the last month? (2) How many times did you receive inpatient care during the past year? In this study, healthcare utilization was considered a continuous variable.

### Independent variables

Our study divided independent variables into health need factors and socioeconomic factors. The independent variables were converted into categorical variables and digitally coded. The proxies of the health need factors were age, gender and self-rated health status (SRH). Age was calculated from the interview dates and self-reported birth date and categorized into three levels (60 − 69 = 1, 70 − 79 = 2, 80 +  = 3). Gender included male and female (male = 1, female = 2). SRH was obtained by asking participants to respond to the question “How would you rate your health status?”, and it was divided into five levels in the questionnaire of CHARLS (very good = 1, good = 2, fair = 3, poor = 4, very poor = 5). Socioeconomic factors were represented by education, marital status, number of children, residence, insurance coverage and per capita household outcome. The first four independent variables were defined as binary variables. The specific encoding is education (junior high school or below = 1, senior high school or above = 2), marital status (partnered = 1, single = 2), number of children (≤ 2 = 1, ≥ 3 = 2) and residence (rural = 1, urban = 2). Moreover, the other independent variables were defined as multi-category variables. Insurance coverage was categorized into three levels (the Urban Employee Basic Medical Insurance (UEBMI) = 1, the Urban and Rural Resident Basic Medical Insurance (URRBMI)/the Urban Residents Basic Medical Insurance (URBMI)/the New Rural Cooperative Medical Scheme (NRCMS) = 2, others = 3). The per capita household outcome of the respondents was ranked from high to low and divided into five levels by the method of quintile (lowest = 1, second = 2, third = 3, fourth = 4, highest = 5).

Given that our study focused on income-related healthcare utilization inequity in the elderly with chronic disease, economic status is an important independent variable. Hence, following recent research [40], we chose to use per capita household consumption to quantify economic status. The data on the per capita household consumption were obtained directly from the Harmonised CHARLS database, which is a user-friendly version of a subset of the CHARLS interviews, to increase the accessibility of the data to researchers and facilitate comparisons [41].

### Statistical method

First, we used LCA on all samples to identify the potential classes of the elderly with chronic diseases. LCA allows for model-based clustering of heterogeneous populations and helps investigators determine if unmeasured or unobserved groups exist within a population. Different from other methods of clustering, LCA permits objective testing of model fit [42]. We comprehensively considered the Akaike information criterion (AIC), Bayesian information criterion (BIC), adjusted Bayesian information criterion (aBIC) and entropy index to determine the number of latent classes, which are indices of how well a model fit. The smaller the values of these indexes, the better the model fits. Further, if the percentage of latent class was less than 10%, it could represent chance findings and be a false indication of the optimal number of latent classes [43].

Second, the differences, including differences in dependent variables and independent variables, between latent classes were examined through analysis of variance and ${x}^{2}$ test, the former is used for continuous variables and the latter for categorical variables.

Third, the CI was calculated to quantify the total inequities in healthcare utilization [44]. The CI ranged from − 1 to 1. The CI equal to zero is indicative that there is no inequity; the positive CI is indicative of the disproportionate concentration of healthcare utilization in rich individuals and the negative CI is indicative of the disproportionate concentration of healthcare utilization in poor individuals [45]. The formula for CI can be written as follows:

$$C = \frac{2}{\mu } {cov}_{w}({y}_{i} , {r}_{i})$$

where $C$ is the CI;$\mu$ is the (weighted) mean healthcare utilization; ${cov}_{w}$ is the weighted covariance and ${y}_{i}$ and ${r}_{i}$ are the healthcare utilization and the scoring rank of the per capita household outcome of an individual $i$($i$=1 for the poorest and $i$=N for the richest), respectively [46].

Fourth, the CI is further decomposed into the contributions of health need factors and socioeconomic factors [12, 46]. Multiple linear regression was used in this study, given that the frequency of healthcare utilization, the dependent variable, is a continuous variable:

$${y}_{i} = \alpha +{\sum }_{v}{\beta }_{v}{x}_{vi}+{\sum }_{j}{\beta }_{j}{x}_{ji}+{\varepsilon }_{i}$$

where ${y}_{i}$ is the frequency of healthcare utilization, ${x}_{vi}$ represents the health need factors, ${x}_{ji}$ represents the socioeconomic factors, ${\beta }_{v}$ and ${\beta }_{j}$ are the marginal effects of each variable and ${\varepsilon }_{i}$ is the error term.

The decomposition of the total CI can be written as follows:

$$C = \alpha +{\sum }_{v}\frac{{\beta }_{i}{\overline{x} }_{i}}{\mu }{C}_{y}+{\sum }_{j}\frac{{\beta }_{j}{\overline{y} }_{j}}{\mu }{C}_{j}+\frac{G{C}_{\varepsilon }}{\mu }$$

where $C$ denotes the total CI; ${\sum }_{v}\frac{{\beta }_{i}{\overline{x} }_{i}}{\mu }{C}_{y}$ is the contribution of the health need factors, ${\sum }_{j}\frac{{\beta }_{j}{\overline{y} }_{j}}{\mu }{C}_{j}$ is the contribution of the socioeconomic factors and $\frac{G{C}_{\varepsilon }}{\mu }$ is the contribution of $\varepsilon$. In this study, health need factors include age, gender and SRH, while socioeconomic factors include education, marital status, number of children, residence, insurance coverage and per capita household outcome.

Fifth, HI indicates the inequity of healthcare utilization in individuals with equal health demands. Wagstaff proposed calculating CI as CI minus the contribution of health need factors [46] with the formula:

$$HI = C-{\sum }_{v}\left(\frac{{\beta }_{i}{\overline{y} }_{i}}{\mu }\right){C}_{y}$$

The range of HI is (− 1, 1), and the meaning of the value is similar to that of CI. A value close to zero is indicative of low inequity. A value within (− 1, 0) indicates pro-poor inequity, and that within (0, 1) indicates pro-rich inequity.

We used MPLUS8.1 (Muthen & Muthen, Los Angeles, U.S.) to perform LCA. Other analyses were performed with Stata, version 13.0 (StataCorp LLC), and significance was set at *p* < 0.05.

## Ethics statement

Data collection from CHARLS received ethical approval from the Institutional Review Board (IRB) of Peking University. The IRB approval number for the main household survey, including anthropometrics, is IRB00001052-11015. Respondents were asked to sign two copies of the informed consent in the survey.

## Results

Table 1 shows the model fitting results of the LCA for patients with chronic disease. The Class7 solution is considered to be unacceptable given the presence of a group with less than 10% of the entire sample. A very small class may present accidental discoveries and provide incorrect indications. Therefore, given its small BIC, aBIC and AIC and entropy score close to 1, Class5 is the most optimal subgroup.

**Table 1:** Model fit indices for LCA

	AIC	BIC	aBIC	Entropy	Conditional probabilities
Class1	85292.857	85389.895	85345.406		
Class2	84126.507	84327.514	84235.358	0.695	88%/12%
Class3	83075.397	83380.374	83240.551	0.542	47%/43%/10%
Class4	82545.988	82954.934	82767.444	0.583	37%/42%/11%/10%
Class5	82209.317	82722.233	82487.076	0.715	20%/10%/13%/12%/44%
Class6	82050.480	82667.364	82384.541	0.699	11%/19%/13%/35%/10%/12%
Class7	81870.058	82590.912	82260.422	0.730	14%/21%/10%/14%/12%/22%/8%

**Fig.1:**
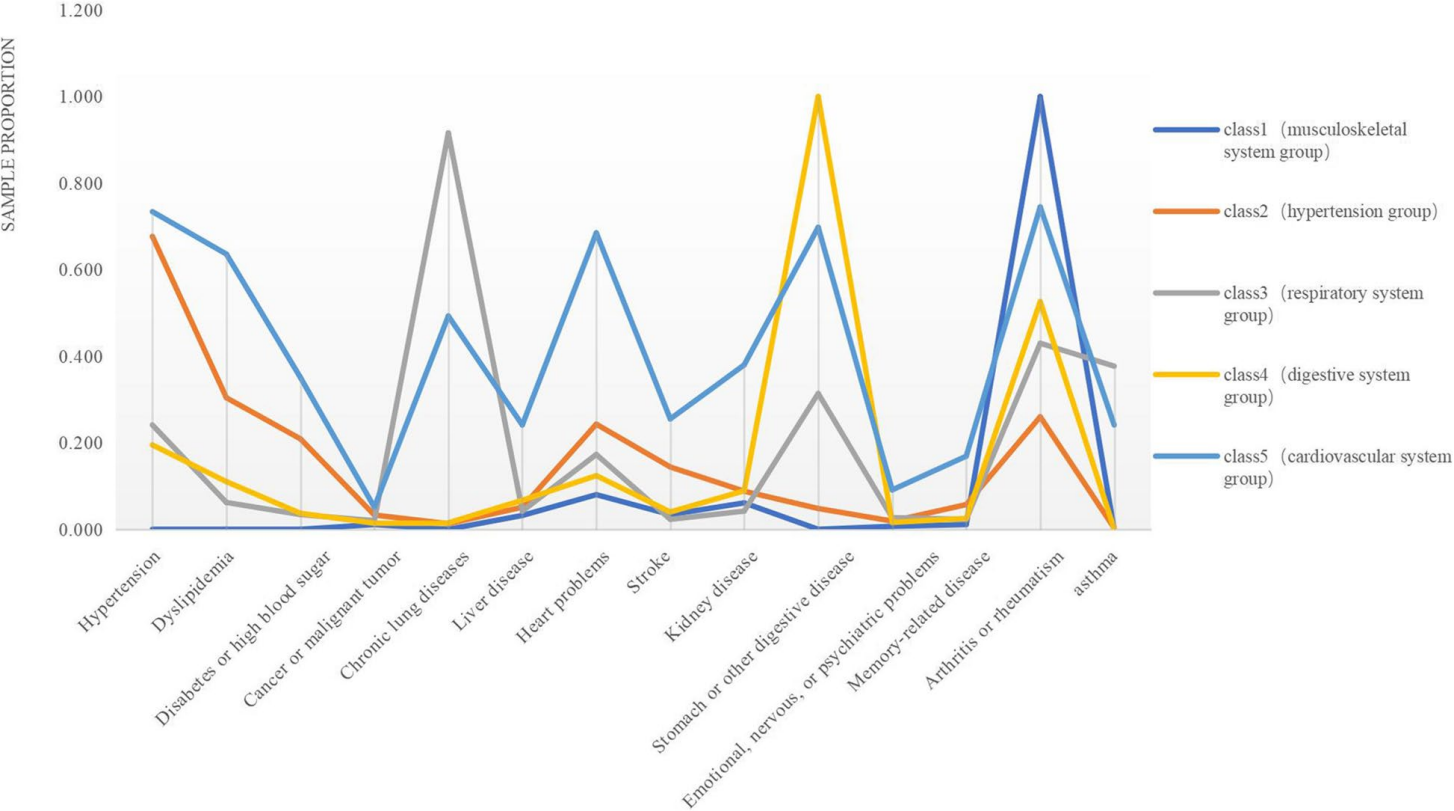
Distribution of chronic diseases in different latent classes

Figure 1 shows the distribution of chronic diseases in latent classes after determining the most optimal number of classifications. We named each latent class based on the prevalence of chronic disease in the five classes, taking into account the characteristics of the diseases with high prevalence in each group. Class1 is named the musculoskeletal system group because 100% of the patients in this class have arthritis or rheumatism. Class2 is designated as the hypertension group because hypertension has the highest prevalence in this group and accounts for 67.7% of the cases. Class3 is called the respiratory system group because 91.6% of the patients in this group have chronic lung diseases. Class 4 is named the digestive system group because it has a high percentage of patients with stomach or other digestive diseases. Four diseases account for more than 50% of the total patients in Class 5: hypertension (73.4%), heart problems (68.5%), stomach or other digestive diseases (69.8%) and arthritis or rheumatism (74.5%). Therefore, Class5 is designated as the cardiovascular system group.

Table 2 shows the social demographic characteristics of the respondents. A total of 7,565 patients over 60 years of age with chronic diseases are enrolled in the study. The majority, or 90.8% of the respondents are aged 60 − 79 years old. The sex ratio is almost even, with 48.0% males and 52.0% females. More than half of the respondents have partners and three or more children. In addition, 91.0% of the respondents have an educational attainment of secondary education, and 94.5% of the respondents are enrolled in basic medical insurance. The sample sizes of the five latent classes are 1506, 784, 977, 937 and 3361, respectively. The differences in social demographic characteristics among the five latent classes are statistically significant (*P* < 0.05). Patients in the respiratory system group are older and more likely to be male than those in other groups. Patients in the hypertension group have worse SRH than others. Patients in the digestive system group are characterized by lower educational and economic levels than those in other groups. The average frequencies of the outpatient visits of the hypertension group (*n* = 0.668 ± 0.057) and musculoskeletal (*n* = 0.463 ± 0.036) system group are higher than those of all other respondents (*n* = 0.423 ± 0.017). The frequencies of inpatient visits of the hypertension group (*n* = 0.804 ± 0.048) and respiratory system group (*n* = 0.536 ± 0.034) are higher than the average level (*n* = 0.414 ± 0.012).

**Table 2:** Social demographic characteristics of the respondents

Variables	All *N* = 7565	Latent class					*P*
		Musculoskeletal system group	Hypertension group	Respiratory system group	Digestive system group	Cardiovascular system group	
		*N* = 1506	*N* = 784	*N* = 977	*N* = 937	*N* = 3361	
**Dependent variables**							
Frequency of one-month outpatient visit, mean (SD)	0.423 (0.017)	0.463 (0.036)	0.668 (0.057)	0.419 (0.046)	0.279 (0.029)	0.390 (0.029)	< 0.05
Frequency of one-year inpatient visit, mean (SD)	0.414 (0.012)	0.344 (0.027)	0.804 (0.048)	0.536 (0.034)	0.202 (0.022)	0.378 (0.018)	< 0.05
**Health need factors**							
Age							< 0.05
60 − 69	4467 (59.0%)	949 (63.0%)	449 (57.3%)	525 (53.7%)	582 (62.1%)	1962 (58.4%)	
70 − 79	2408 (31.8%)	454 (30.1%)	266 (33.9%)	335 (34.3%)	274 (29.2%)	1079 (32.1%)	
≥ 80	69 0 (9.2%)	103 (6.8%)	69 (8.8%)	117 (12.0%)	81 (8.6%)	320 (9.5%)	
Gender							< 0.05
Male	3629 (48.0%)	682 (45.3%)	327 (41.7%)	573 (58.6%)	405 (43.2%)	1642 (48.9%)	
Female	3936 (52.0%)	824 (54.7%)	457 (58.3%)	404 (41.4%)	532 (56.8%)	1719 (51.1%)	
SRH							< 0.05
Very good	535 (7.1%)	91 (6.0%)	17 (2.2%)	50 (5.1%)	89 (9.5%)	288 (8.6%)	
Good	717 (9.5%)	139 (9.2%)	26 (3.3%)	83 (8.5%)	116 (12.4%)	353 (10.5%)	
Fair	3571 (47.2%)	751 (49.9%)	261 (33.3%)	426 (43.6%)	475 (50.7%)	1658 (49.3%)	
Poor	2092 (27.7%)	409 (27.2%)	328 (41.8%)	318 (32.5%)	191 (20.4%)	846 (25.2%)	
Very poor	650 (8.6%)	116 (7.7%)	152 (19.4%)	100 (10.2%)	66 (7.0%)	216 (6.4%)	
**Socioeconomic factors**							
Education							< 0.05
Junior high school or below	6883 (91.0%)	1380 (91.6%)	715 (91.2%)	922 (94.4%)	897 (95.7%)	2969 (88.3%)	
Senior high school or above	682 (9.0%)	126 (8.4%)	69 (8.8%)	55 (5.6%)	40 (4.3%)	392 (11.6%)	
Marital status							< 0.05
Partnered	5781 (76.4%)	1163 (77.2%)	576 (73.5%)	722 (73.9%)	698 (74.5%)	2622 (78.0%)	
Single	1784 (23.6%)	343 (22.8%)	208 (26.5%)	255 (26.1%)	239 (25.5%)	739 (22.0%)	
Number of children							< 0.05
≤ 2	3168 (41.9%)	650 (43.2%)	302 (38.5%)	363 (37.2%)	349 (37.2%)	1504 (44.7%)	
≥ 3	4397 (58.1%)	856 (56.8%)	482 (61.5%)	614 (62.8%)	588 (62.8%)	1857 (55.3%)	
Residence							< 0.05
Rural	4677 (61.8%)	1004 (66.7%)	483 (61.6%)	669 (68.5%)	650 (69.4%)	1871 (55.7%)	
Urban	2888 (38.2%)	502 (33.3%)	301 (38.4%)	308 (31.5%)	287 (30.6%)	1490 (44.3%)	
Insurance coverage							< 0.05
UEBMI	1111 (14.7%)	173 (11.5%)	124 (15.8%)	97 (9.9%)	76 (8.1%)	641 (19.1%)	
URRBMI/URBMI/NRCMS	6035 (79.8%)	1249 (82.9%)	621 (79.2%)	834 (85.4%)	804 (85.8%)	2527 (75.2%)	
Others	419 (5.5%)	84 (5.6%)	39 (5.0%)	46 (4.7%)	57 (6.1%)	193 (5.7%)	
Per capita household outcome							< 0.05
Lowest	1513 (20.0%)	300 (19.9%)	120 (15.3%)	223 (22.8%)	252 (26.9%)	618 (18.4%)	
Second	1513 (20.0%)	301 (20.0*)	132 (16.8%)	206 (21.1%)	208 (22.2%)	666 (19.8%)	
Third	1513 (20.0%)	321 (21.3%)	171 (21.8%)	185 (18.9%)	172 (18.4%)	664 (19.8%)	
Fourth	1513 (20.0%)	297 (19.7%)	177 (22.6%)	175 (17.9%)	164 (17.5%)	700 (20.8%)	
Highest	1513 (20.0%)	287 (19.1%)	184 (23.5%)	188 (19.2%)	141 (15.0%)	713 (21.2%)	

Tables 3 and 4 show the inequity and the decomposition of the frequencies of the outpatient and inpatient visits in the five latent classes. The CI reports the total healthcare inequity. On the whole, the CI of the frequency of outpatient visits is 0.080 and that of the frequency of inpatient visits is 0.135. Both CI values are positive and indicate the tendency for pro-rich inequity. For latent classes, the CI of most groups are positive for outpatient visits or inpatient visits, but only the CI of the musculoskeletal system group in an outpatient visit is negative (CI = -0.037), indicating that healthcare utilization is concentrated in the poor. Numerically, for the frequency of outpatient visits, the CI values of the digestive system group (CI = 0.197) and cardiovascular system group (CI = 0.143) are higher than the overall level and the CI of the respiratory system group (CI = 0.001) is the smallest. For the frequency of inpatient visits, the CI of the digestive system group (CI = 0.157) and musculoskeletal system group (CI = 0.145) are larger than the overall value and that of the respiratory system group (CI = 0.086) is the lowest.

**Table 3:** Contribution of each independent variable to the inequity in one-month outpatient utilization

Variables	Musculoskeletal system group		Hypertension group		Respiratory system group		Digestive system group		Cardiovascular system group		All	
	cont	%	cont	%	cont	%	cont	%	cont	%	cont	%
**CI**	** − 0.037**		**0.041**		**0.001**		**0.197**		**0.143**		**0.080**	
**Health need factors**												
Age (Ref: 60 − 69)		0.002		8.728		− 760.35		10.784		8.545		4.961
70 − 79	0.000	− 0.304	0.004	8.998	− 0.003	− 259.337	− 0.00	− 0.427	0.003	2.361	0.001	1.730
≥ 80	0.000	0.306	0.000	− 0.270	− 0.005	− 501.013	0.022	11.211	0.009	6.184	0.003	3.231
Gender (Ref: Male)	− 0.007	19.972	− 0.003	− 6.863	0.012	1166.393	0.031	15.896	− 0.003	− 1.911	0.002	3.099
SRH (Ref: Very good)		44.915		59.605		− 1422.284		81.657		62.908		57.167
Good	0.007	− 19.563	0.002	5.020	0.005	495.939	− 0.003	− 1.675	0.000	0.207	0.001	0.966
Fair	− 0.002	6.703	0.010	24.591	− 0.015	− 1497.38	0.094	47.925	0.069	48.509	0.034	42.048
Poor	− 0.014	36.503	0.015	35.385	− 0.001	− 53.862	0.032	16.475	0.014	10.063	0.010	12.872
Very poor	− 0.008	21.272	− 0.002	− 5.391	− 0.004	− 366.981	0.037	18.932	0.006	4.129	0.001	1.281
**Add up**	− **0.024**	**64.889**	**0.026**	**61.47**	− **0.011**	− **3349.027**	**0.212**	**108.337**	**0.098**	**69.542**	**0.052**	**65.227**
**Socioeconomic factors**												
Education (Ref:Junior high school or below)	0.000	− 0.870	− 0.001	− 3.425	− 0.001	− 79.647	0.005	2.784	− 0.001	− 0.831	0.002	2.337
Marital status (Ref: Partnered)	− 0.004	11.654	− 0.006	− 15.278	− 0.001	98.121	− 0.017	− 8.418	0.001	0.457	− 0.002	− 1.881
Number of children(Ref: ≤ 2)	0.014	− 37.794	− 0.002	− 4.691	− 0.002	− 249.641	− 0.011	− 5.445	− 0.004	− 2.529	− 0.001	− 1.720
Residence (Ref: Rural)	− 0.000	0.553	− 0.006	− 14.095	0.000	32.748	− 0.017	− 8.707	− 0.009	− 6.068	− 0.009	− 10.899
Insurance coverage (Ref: UEBMI)		10.665		5.57		1027.572		− 31.032		− 14.457		− 13.125
URRBMI/URBMI/NRCMS	− 0.004	11.319	− 0.001	− 3.332	0.009	881.006	− 0.057	− 29.001	− 0.015	− 10.618	− 0.007	− 8.212
Others	0.000	− 0.654	0.004	8.902	0.001	146.566	− 0.004	− 2.031	− 0.005	− 3.839	− 0.004	− 4.913
Per capita household outcome (Ref: Lowest)		− 9.58		− 2.417		2030.291		34.787		− 1.007		3.514
Second	0.005	− 12.337	0.001	3.035	0.029	2915.396	0.004	1.965	0.000	− 0.234	0.000	− 0.346
Third	− 0.004	9.676	0.000	− 0.644	− 0.004	− 385.046	0.013	6.582	− 0.004	− 2.657	0.002	3.021
Fourth	0.001	− 3.139	− 0.001	− 2.903	− 0.007	− 740.137	0.046	23.177	0.000	− 0.073	0.000	0.527
Highest	0.001	− 3.780	− 0.001	− 1.905	0.002	240.078	0.006	3.063	0.003	1.957	0.000	0.312
**Add up**	**0.009**	** − 25.372**	** − 0.013**	** − 34.336**	**0.026**	**2859.444**	** − 0.032**	** − 16.031**	** − 0.034**	** − 24.435**	** − 0.019**	** − 21.774**
**HI**	** − 0.013**		**0.015**		**0.012**		** − 0.015**		**0.045**		**0.028**

**Table 4:** Contributions of each independent variable to the inequity in one-year inpatient utilization

Variables	Musculoskeletal system group		Hypertension group		Respiratory system group		Digestive system group		Cardiovascular system group		All	
	cont	%	cont	%	cont	%	cont	%	cont	%	cont	%
**CI**	**0.145**		**0.128**		**0.086**		**0.157**		**0.120**		**0.135**	
**Health need factors**												
Age (Ref: 60 − 69)		13.445		9.032		6.16		47.353		12.623		13.702
70 − 79	0.008	5.808	0.006	4.337	0.005	5.242	0.054	34.456	0.014	11.553	0.014	10.146
≥ 80	0.011	7.637	0.006	4.695	0.001	0.918	0.020	12.897	0.001	1.070	0.005	3.556
Gender (Ref: Male)	− 0.009	− 6.156	− 0.016	− 12.653	− 0.015	− 16.938	− 0.019	− 12.028	− 0.013	− 10.645	− 0.016	− 11.508
SRH (Ref: Very good)		97.295		73.399		77.524		84.431		71.987		79.399
Good	− 0.003	− 1.810	0.000	− 0.139	− 0.001	− 0.608	0.052	33.237	− 0.008	− 6.347	− 0.002	− 1.310
Fair	0.015	10.179	0.019	14.699	0.014	16.410	0.049	31.390	0.024	20.027	0.025	18.193
Poor	0.090	61.922	0.052	40.590	0.016	19.153	− 0.010	− 6.076	0.053	44.128	0.054	39.997
Very poor	0.039	27.004	0.023	18.249	0.037	42.569	0.041	25.880	0.017	14.179	0.030	22.519
**Add up**	**0.151**	**104.584**	**0.09**	**69.778**	**0.057**	**66.746**	**0.187**	**119.756**	**0.088**	**73.965**	**0.11**	**81.593**
**Socioeconomic factors**												
Education (Ref: Junior high school or below)	0.000	0.341	− 0.002	− 1.764	0.001	0.875	− 0.009	− 5.493	− 0.002	− 1.606	− 0.001	− 0.990
Marital status (Ref: Partnered)	0.002	1.118	0.006	4.742	0.001	0.679	− 0.021	− 13.319	− 0.002	− 1.530	0.000	0.312
Number of children(Ref: ≤ 2)	0.003	1.793	0.012	9.634	0.039	45.407	0.009	5.555	0.016	13.163	0.018	13.096
Residence (Ref: Rural)	0.004	2.414	0.009	6.955	− 0.004	− 5.055	0.004	2.496	0.008	6.353	0.007	4.983
Insurance coverage (Ref: UEBMI)		− 7.188		− 3.747		9.202		66.663		− 7.01		− 4.505
URRBMI/URBMI/NRCMS	− 0.005	− 3.665	− 0.004	− 3.321	0.008	9.258	0.090	57.171	− 0.008	− 6.842	− 0.005	− 3.398
Others	− 0.005	− 3.523	− 0.001	− 0.426	0.000	− 0.056	0.015	9.492	0.000	− 0.168	− 0.001	− 1.107
Per capita household outcome (Ref: Lowest)		33.287		7.885		0.085		19.41		9.42		10.811
Second	0.007	4.775	0.000	− 0.090	0.000	0.158	− 0.003	− 2.046	0.000	0.126	0.000	0.354
Third	0.034	23.523	− 0.002	− 1.621	− 0.002	− 2.179	0.002	1.583	0.002	1.491	0.005	3.887
Fourth	− 0.001	− 0.590	0.001	1.157	0.002	1.977	0.002	1.536	0.004	3.102	0.003	1.909
Highest	0.008	5.579	0.011	8.439	0.000	0.129	0.029	18.337	0.006	4.701	0.006	4.661
**Add up**	**0.047**	**31.765**	**0.03**	**23.705**	**0.045**	**51.193**	**0.118**	**75.312**	**0.024**	**18.79**	**0.032**	**23.707**
**HI**	** − 0.006**		**0.038**		**0.029**		** − 0.030**		**0.032**		**0.024**	

We then decomposed the CI to further determine the contribution of independent variables to healthcare utilization inequity. Independent variables are divided into health need and socioeconomic factors. For the all respondents, the contributions of health need factors to outpatient visit inequity and inpatient visit inequity are 65.227% and 81.593%, respectively, and are larger than the contributions of − 21.774% and 23.707% provided by socioeconomic factors. These results indicate that socioeconomic factors promote the increased use of outpatient services by poor patients. For the frequency of outpatient visits, the top three contributing factors are SRH, insurance coverage and residence with the contribution of 57.167%, − 13.125% and − 10.899%, respectively. For the frequency of inpatient visits, the top three contributing factors are SRH, number of children and age with the contribution of 79.399%, 13.702% and 13.096%, respectively. The results for all latent classes are similar to the overall results, indicating that health need factors have greater contributions than socioeconomic factors. Health need factors provide a pro-poor contribution to the frequency of outpatient visits in the musculoskeletal system group (64.889%) and respiratory system group (-3349.027%), but provide a pro-rich contribution to the frequency of inpatient visits in all latent classes. For all latent classes, socioeconomic factors provide pro-poor contributions to outpatient visits and pro-rich contributions to inpatient visits. Notably, SRH is the major contributor among all independent variables. Moreover, the influence of other independent variables varies among latent classes.

HI indicates the socioeconomic-related inequity in healthcare utilization among individuals with equal health needs. For all respondents, the value of outpatient visits is 0.028 and that of inpatient visits is 0.024, indicating the presence of pro-rich inequity in healthcare utilization. For the frequency of outpatient visits, the HI of the cardiovascular system group (HI = 0.045) is higher than the overall value (HI = 0.028), and the HI values of the musculoskeletal system group (HI =  − 0.013) and digestive system group (HI =  − 0.015) are negative, indicating pro-poor inequity. For the frequency of inpatient visits, only the musculoskeletal system group (HI = -0.006) has an absolute value of HI below the average. In addition, the HI values of the hypertension group (HI = 0.038), respiratory system group (HI = 0.029) and cardiovascular system group (HI = 0.032) are positive. The HI values of the musculoskeletal system group (HI =  − 0.006) and digestive system group (HI =  − 0.030) are negative.

## Discussion

The above results show that elderly patients with chronic diseases are divided into five latent classes with statistically significant different socio-demographic characteristics and different healthcare utilization inequities. According to the CI value, pro-rich inequity in healthcare utilization is found for all respondents and is also the main feature in latent classes except the outpatient visits in the musculoskeletal system group. The digestive system group has the worst equity for access to healthcare utilization and the respiratory system group has the best. After balancing the influence of health need factors, the HI value is closer to zero than the CI value in all latent classes, which means healthcare utilization inequity was alleviated, but not in the respiratory system group for outpatient visit. Notably, in the hypertension group, respiratory system group and cardiovascular system group, there was pro-rich inequity in healthcare utilization, but in the musculoskeletal system group and digestive system group, there was pro-poor inequity. In addition, the decomposition of the CI shows that in all latent classes, the contribution of health need factors is larger than that of socioeconomic factors, and the SRH is the major contributor. Based on the above evidence, we deduce that the economic burden of chronic diseases, residents’ economic affordability and effective reimbursement rate of medical insurance, the effect of chronic diseases on health-related quality of life (HRQL) and the cognition of self-health could be potential factors for the disparities in healthcare utilization inequity among latent classes.

First, the economic burden of chronic diseases may be one of the causes of healthcare utilization inequity because a high economic burden is an important economic obstacle to accessing healthcare services for patients with chronic diseases [11]. Patients with chronic diseases tend to have long-term and expensive healthcare need and low-income patients can often ill afford them. Thus, low-income patients have lower rates of healthcare utilization than affluent patients, and are more likely to not use the healthcare that they need or opt for cheaper healthcare services [47–50]. For example, studies have shown that the medical expenditure of inpatient visits is considerably higher than that of outpatient visits, and thus, low-income patients are more likely to use outpatient than inpatient visits [25, 51, 52]. This conclusion is also reflected in our study, which shows that socio-economic factors prompt low-income patients to use more outpatient visits and high-income patients to use more inpatient visits generally. In addition, pro-rich inequity of healthcare utilization is also more likely to occur in chronic diseases with high medical expenditure. In Japan, kidney diseases, stroke and neurological diseases are most likely to lead to high medical expenditure, whereas chronic respiratory diseases are less associated with high medical expenditure than other diseases [53]. In Mexico, the top four most expensive chronic diseases are chronic kidney disease, arterial hypertension, type 2 diabetes and chronic ischemic heart disease accounting for more than 85% of the total financial burden of chronic diseases [54]. These findings are consistent with our results, the latent classes of these chronic diseases undergo greater pro-rich inequity in healthcare utilization.

Second, the inequity in residents’ economic affordability and effective reimbursement rate of medical insurances is related to the healthcare utilization inequity [55, 56]. The Chinese government established the UEBMI in 1998, the NRCMS in 2002 and the URBMI in 2007, which constituted China's basic social medical insurance system and covered 95% of the population [57]. To provide the same service coverage and financial protections, the URRBMI, which is based on the NRCMS and the URBIM, was gradually implemented nationwide in 2016 [5]. Theoretically, medical insurance can reduce healthcare utilization inequity because of financial support. However, a review of the literature revealed that medical insurance has intensified the inequity between the UEBMI and the URRBMI [25, 58]. The possible reasons are the UEBMI has a higher reimbursement rate, and bigger benefits packages than the URRBMI. In addition, the individuals covered by the UEBMI have formal jobs and stronger economic capability [25, 59]. Thus, individuals covered by UEBMI are more likely to access health services or even overuse them.

Third, the effects of chronic diseases on HRQL may lead to disparities in healthcare utilization inequity. HRQL is a fairly broad, multidimensional concept that addresses the domains of physical, mental, emotional and social functioning, and a core component of its definition is the focus on the individual’s subjective feelings of quality of life [60, 61]. The decline in physical and mental health may be a driver of healthcare utilization. The symptoms of diseases that impede the ability to perform their tasks for daily living may compel patients to seek healthcare, and allow patients to ignore other restrictions [51, 52, 62]. On the one hand, patients are more likely to use healthcare based on their health needs when chronic diseases have a worse effect on quality of life, and healthcare utilization equity will be better. Current studies have shown that certain chronic diseases, such as hypertension, asthma, arthritis and chronic obstructive pulmonary disease, may have a more undesirable effect on patients’ HRQL than other diseases [36, 60]. In our study, the healthcare utilization equity for these chronic diseases is better than that for others. On the other hand, if chronic diseases have a great effect on the HRQL and limit the ability to work, patients who need to work will use more health services to alleviate the effects of the disease on work, resulting in healthcare utilization inequity. Low-income seniors usually have fewer social security benefit and need to keep working to sustain their life [63]. This view is also reflected in this study. The digestive system group and musculoskeletal system group have pro-poor healthcare utilization inequity after balancing health need factors, which are more likely to reduce patients’ physical activity [64].

Fourth, the cognition of self-health may influence healthcare utilization inequity. The SRH reflects an individual’s sensitive and holistic view of overall health, including physical and mental health [65, 66]. Low SRH may be associated with increased healthcare utilization [66, 67]. SRH can represent the true health status but is also affected by factors other than health status [66]. Aging is important for SRH. Scholars have found that the elderly tend to ignore their own seemingly minor health problems and overestimate their health status because they regard poor health and functional limitations as the norm [68]. Moreover, the patients’ subjective evaluation of diseases affects their SRH. Compared with life-threatening diseases (such as coronary disease and cancer) patients with debilitating but non-life-threatening diseases (such as osteoarthritis and hypertension) are more likely to overestimate their health status and change their health-related behaviors [66]. Teenagers who do not believe that diabetes can have unhealthy consequences are more likely to drop treatment than those who do not [69]. Finally, studies have shown that an individual’s other characteristics can also affect their SRH. Individuals tend to have a better cognition of self − health when they have higher educational and economic levels although they may have worse health status [66, 70]. Under the combined effect of various factors, people may not be able to judge their health level correctly. Such a situation results in unreasonable medical treatment behavior. In this study, we found that SRH is the major independent variable of healthcare utilization inequity. this result indicates that self-health cognition plays a decisive role in healthcare utilization by the elderly with chronic diseases, especially those in the musculoskeletal system group and respiratory system groups. Thus, the control the gap between SRH and the real health status to a reasonable level and the prevention of the delayed treatment or over-treatment of diseases are important to mitigate healthcare utilization inequity.

This study has several limitations. Firstly, it is a cross-sectional study that compares healthcare utilization inequity among latent classes, Therefore, we did not observe how inequity indicators change over time and did not determine any potential causal association. Future research will measure the healthcare utilization inequity at multiple points in time. Such an approach helps observe and understand the changing trend of indicators. Second, chronic diseases and healthcare utilization are reported by the respondents and lack medical evidence. This situation could lead to recall bias. Self-reported data may have under-reporting or over-reporting problems that influence the final results. However, recall bias exists in all retrospective studies and is unavoidable. The literature shows that self-reported measures of chronic diseases have reasonable accuracy and are widely used [71, 72]. Third, due to the data limitation, we only considered the occurrence of chronic diseases under investigation and ignored their duration or severity in the classification of patients with chronic diseases through LCA. We will be able to flesh out our research if we obtain such data in the future.

## Conclusion

This paper shows that pro-rich is still the main manifestation of health service utilization inequity in elderly patients with chronic diseases, and the features of inequity vary among latent classes. Moreover, the health need factors contribute more than socioeconomic factors, and the SRH is the most important factor leading to healthcare utilization inequity. According to the results, we think the healthcare utilization inequity and its differences among latent classes may be associated with the economic burden of chronic diseases, residents’ economic affordability and effective reimbursement rate of medical insurances, HRQL and self-health cognition. Therefore, the government needs to provide financial support and expanded the benefits packages of basic medical insurance, which is based on a basic social medical insurance system for the whole population, to low-income patients suffering from chronic diseases related to high medical expenditure to enhance the equity of healthcare utilization by elderly patients. Moreover, the government and healthcare providers should pay attention to the physical and mental feelings of elderly patients with chronic diseases and guide them to evaluate their health status correctly to promote reasonable access to health care.

## Data Availability

The datasets analysed during the current study are available on http://charls.pku.edu.cn/pages/data/2015-charls-wave4/en.html.

## Abbreviations

CI: Concentration index
SRH: Self-rated health status
LCA: Latent class analysis
HI: Horizontal index
CHARLS: China Health and Retirement Longitudinal Study
AIC: Akaike information criterion
BIC: Bayesian information criterion
aBIC: Adjusted Bayesian information criterion
IRB: Institutional Review Board
HRQL: Health-related quality of life
NRCMS: New Rural Cooperative Medical Scheme
URBMI: Urban Residents Basic Medical Insurance
URRBMI: Urban and Rural Resident Basic Medical Insurance
